# Impact of Covid-19 pandemic lockdown on the urban litter and clean environment index

**DOI:** 10.1038/s41598-023-35554-1

**Published:** 2023-06-05

**Authors:** Khadijeh Darabi, Ramin Hayati, Maryam Morovati, Navid Alinejad, Ghasem Hassani

**Affiliations:** 1 Department of Environmental Health Engineering, Shiraz University of Medical Sciences, Shiraz, Iran; 2 Department of Public Health, Fasa University of Medical Sciences, Fasa, Iran; 3 Department of Environmental Sciences and Engineering, Faculty of Agriculture and Natural Resources, Ardakan University, Ardakan, Iran; 4 Department of Environmental Health Engineering, School of Health, Yasuj University of Medical Sciences, Yasuj, Iran

**Keywords:** Environmental sciences, Environmental social sciences

## Abstract

Changing the level of pollution in the urban environment is one of the consequences of Covid-19. Litter are one of the most important urban pollutants affected by the Covid-19 pandemic. In this research, the pollution level of urban areas during the Covid-19 pandemic was investigated by studying the urban environment. To this end, the protocol of observation and counting was used and litter were studied in two groups including common litter and Covid-19 related litter in Yasuj, Iran. The results were interpreted based on the clean environment index (CEI). The time of observation was selected based on the peak of the disease and the decline in the incidence rate. The results showed that on average, at the peak of the disease, the density of the litter was reduced by 19% compared to the low lockdown related to Covid-19. The CEI on average was 4.76 at the peak of the disease that was interpreted in the clean status, while the CEI on average was 5.94 at the low lockdown related to Covid-19 so interpreted in the moderate status. Among urban land uses, recreational areas with a difference of more than 60% showed the greatest impact caused by Covid-19, while in commercial areas this difference was less than 3%. The effect of Covid-19 related litter on the calculated index was 73% in the worst case and 0.8% in the lowest case. Although Covid-19 decreased the number of litter in urban areas, the emergence of Covid-19 lockdown related litter was a cause for concern and led to increasing the CEI.

## Introduction

The outbreak of a new species of the coronavirus in 2019 in China was the source of an epidemic disease called COVID-19^1,2^ that recognized as a pandemic by the World Health Organization (WHO) in 2020 after it spread to 114 countries^3^. The effects of the pandemic on the environment quickly became apparent^4,5^. However the COVID-19 pandemic leads to reduction of nitrogen and particulate matter emissions^6^, but it has resulted in adverse and serious consequences for the environment such as increase in plastic consumption^7,8^.

The effect of the pandemic on the composition and quantity of medical wastes and municipal solid wastes is one of the big challenges about the environmental consequences of the pandemic^9,10^. Increasing production of plastic waste, even in the post-pandemic era, must be managed by social responsibility, corporate action, and government policy^11^. Of course, the impact of the pandemic on the quantity of municipal solid waste is not always increasing. These effects are different according to geographical and sociological characteristics, so that during the pandemic, municipal solid waste generation in Shanghai decreased by 23%, but in Singapore it increased by 3%^12^. The Covid-19 pandemic has caused the emergence of new components of municipal solid waste, of which face masks are the most important. During the pandemic, 4214, 310, 558, 122, and 309 tons of the face mask waste are generated daily in China, Turkey, Japan, Malaysia, and Iran, respectively^13^. The change in the quantity and composition of municipal solid waste in the COVID-19 pandemic is due to two important reasons: first, in the epidemic conditions, the lifestyle will change according to the conditions; second, the health needs of society will increase production and consumption in some sectors^14–16^.

The lifestyle of citizens and consumers has a direct and indirect effect on environmental pollution, which has been proven in energy efficiency^17^. Litter is a waste that has not been properly disposed of in trash bins by citizens^18,19^. This behavior causes waste to be scattered in many urban and public environments^20^. Litter can have serious health and environmental consequences, as well as create unfavorable landscapes^21^. It includes various types of municipal solid waste, of which plastic and paper are the most common^22,23^. Aim of this study was to evaluate the impact of the Covid-19 pandemic on the composition and quantity of litter in urban environment. Also, an attempt was made to investigate the direct consequences of the pandemic in the emergence of new litter, as well as the indirect consequences of the disease in the status of urban pollution.

## Method

### Study area

**Figure 1:**
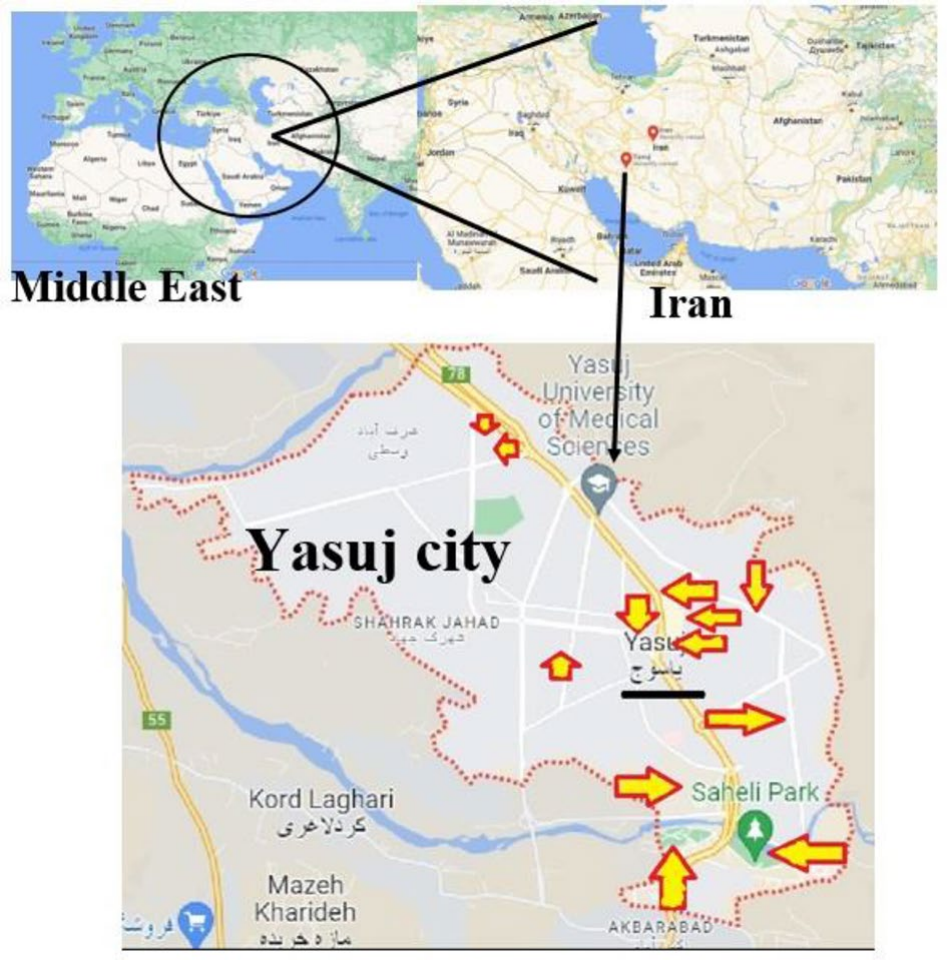
Studied location^24^.

This study was conducted to investigate the density and composition of litter in urban environments in Yasuj, Iran. This city is the center of a province in the south of Iran, which has a population of more than 130,000 people. The climate of Yasuj city is a temperate Mediterranean mountain with mild summers and cold and rainy winters. This study was conducted in different location of the city according to the classification of areas based on the type of land-use (Fig. 1). The study was conducted in 12 locations in the city, including nine streets with different land-uses and three parks (Pirsheh street, Sardar street, Kashani Boulevard, Ferdowsi street, Talegani Boulevard, Ommat Boulevard, Emam Khomeyni Boulevard, Kamaledin street, Saeidi street, Velayat park, Saheli park, and Mehrvarzi park), which were named LA to LL.

### Litter study protocol

Information on the density of litter (item/m^2^) was obtained based on the field method for counting the number of litter using visual surveys^18,25^. In this method, the studied locations were visited by the researchers at certain times of the day, and information was obtained by direct observations^11,19^. The study was based on a specific protocol in the evening of working days^18,26^. The criteria for the investigation of the studied locations was only the number of litter, and the weight and volume of the littered wastes were omitted due to the impact of secondary pollution and intervening conditions such as moisture^27^. The streets were surveyed on both sides. The total width of the sidewalk plus a meter of street depth was determined as the width of the liter count^18,28^. The study was conducted in one year (September 2021–September 2022), during which time each location was visited 12 times (once every month) and data were recorded. At the time of this study, due to the increase in the number of positive cases of Covid-19 and the rate of hospitalizations, the government implemented lockdown twice, which included restrictions on the presence of citizens in the city and reducing the hours of commercial and administrative activities. The first and second lockdown periods lasted about three weeks and four weeks, respectively. According to the one-month interval of litter assessment in each location, two assessments out of a total of twelve assessments were conducted for each location during lockdown.

### Litter study criteria

Littered solid wastes by citizens in the environment was considered litter and items such as tree branches, leaves and broken pieces of sidewalk surface were not considered^23^. In this study, litter were investigated in two general categories, which included common litter (CL) and COVID-related litter (CRL). The CL included items such as plastics, papers, metals, wood, and tobacco wastes. Items such as face masks, gloves, alcoholic solution-based bottles, and face shields were classified as CRL.

### Calculation of pollution status

Information on the quantity of litter at the studied locations was interpreted using the clean environment index (CEI)^29^.

1 $$CEI=\frac{\sum (\mathrm{Wi }\times \mathrm{Ni})}{\mathrm{Lenght }\left(\mathrm{m}\right)\times \mathrm{Width }(\mathrm{m})} \times K$$

**Figure 2:**
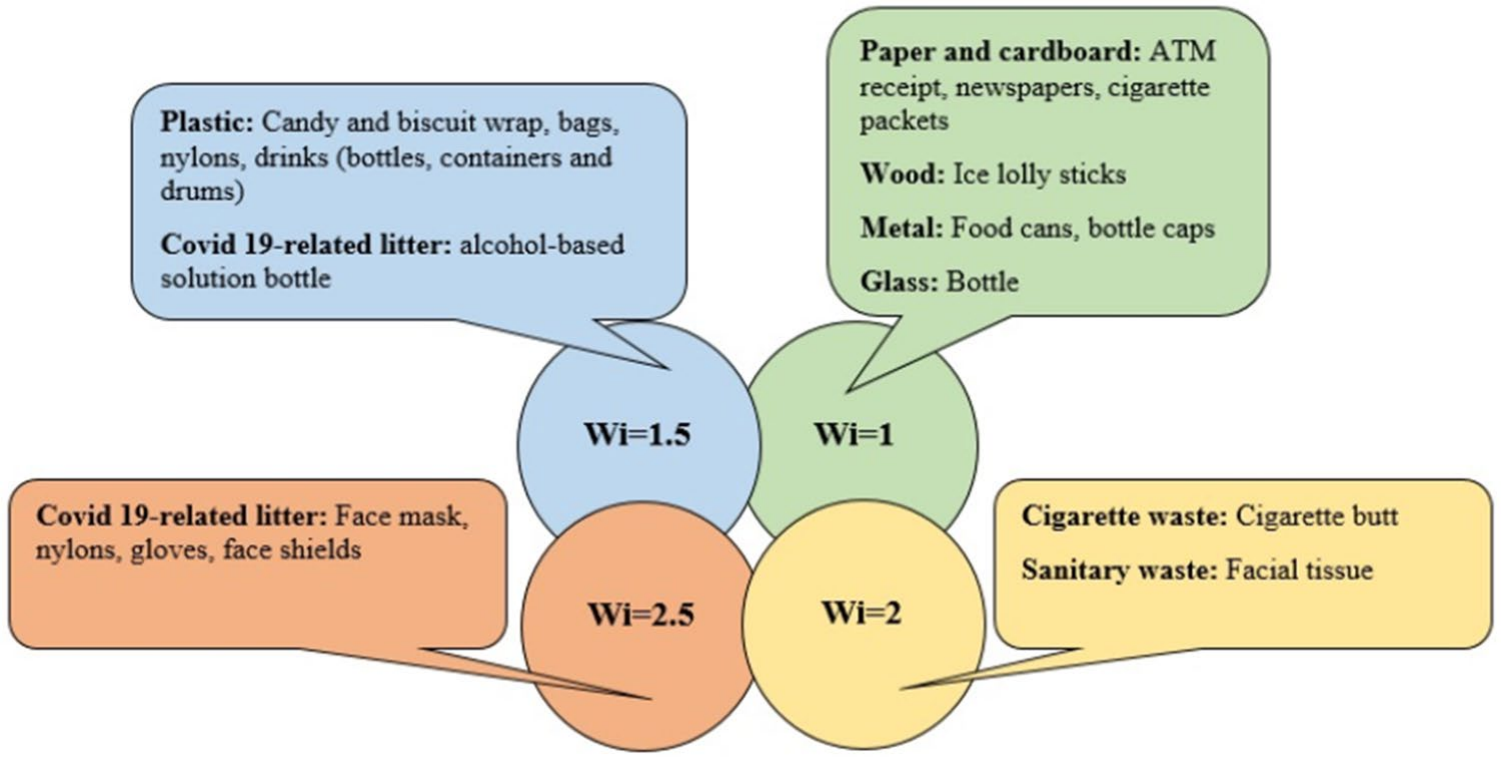
Wi coefficients for litter types^28,29^.

In this formula Ni represents the number of observed litter and K is a constant coefficient equal to 20. The Wi coefficient used in this formula is defined according to the potential for damage of each litter to the environment and health^18,29^. The width in this formula for each location included the entire width of the sidewalk and 1 m from the street. The width in this formula for each location included the length of the street^30^. The Wi coefficient for different categories of litter has been shown in Fig. 2. Some types of CRL were calculated with Wi = 2.5 in the formula due to the possibility of virus infection, such as face masks. But some types of CRL, which are made of plastic and have a low probability of being infected with the virus, were calculated with Wi = 1.5 in the formula^28,29^.

## Results and discussion

The density of litter at the locations studied have been shown in Table 1. The average of observed litter in two lockdown times is stated in the LT section. The average amount of observed litter in ten times without lockdown is stated in the NLT section. Among the locations, the litter density in LG was 0.689 item/m^2^, which was higher than that in other locations. Also, the litter density was at the lowest value equal to 0.0125 item/m^2^, which was observed in LJ and was 54 times lower than the highest litter density in the studied areas. On average, in the areas studied, the litter density was 0.287 items/m^2^. A cigarette butt was the most CL in the urban environment, with an average of 0.150 items/m^2^, consisting of 52.26% of the total urban litter. The CRL in the total studied locations consisted of 0.88–62% of the total litter, which was equal to 0.0011–0.0069 item/m^2^. On average, CRL accounted for 1.49% of total litter.

**Table 1:** Litter density in studied locations (item/m^2^).

Location	CL														CRL	
	Paper and cardboard		Wood		Metal		Glass		Plastic		Cigarette butt		Facial tissue			
	NLT^a^	LT^b^	NLT	LT	NLT	LT	NLT	LT	NLT	LT	NLT	LT	NLT	LT	NLT	LT
LA	0.123	0.097	0.003	0.0014	0.0075	0.0051	0.0004	0.0001	0.108	0.068	0.254	0.212	0.0189	0.0111	0.0045	0.0039
LB	0.006	0.001	0.001	0.0004	0.0004	0.0003	0.0001	0.00004	0.0172	0.0119	0.112	0.103	0.0002	0.00011	0.0012	0.001
LC	0.001	0.0008	0.001	0.0005	0.0001	0.00004	0.0002	0.00008	0.0008	0.0005	0.002	0.0017	0.0001	0.00007	0.0083	0.0071
LD	0.119	0.083	0.002	0.0012	0.0063	0.0028	0.0003	0.00012	0.098	0.057	0.317	0.298	0.0224	0.0163	0.0032	0.0028
LE	0.102	0.067	0.003	0.0018	0.0045	0.0021	0.0003	0.00016	0.121	0.072	0.182	0.149	0.0173	0.0118	0.0041	0.0037
LF	0.005	0.003	0.001	0.0005	0.0002	0.0001	0.0002	0.00008	0.0156	0.0118	0.088	0.069	0.0002	0.00013	0.0011	0.0009
LG	0.131	0.109	0.005	0.0024	0.0038	0.0017	0.0004	0.00019	0.133	0.108	0.411	0.375	0.0142	0.0098	0.0064	0.0059
LH	0.002	0.0013	0.001	0.0006	0.0002	0.0001	0.0005	0.00032	0.0009	0.0006	0.001	0.0008	0.0003	0.00021	0.0071	0.0064
LI	0.108	0.084	0.004	0.0021	0.0066	0.0029	0.0002	0.00007	0.084	0.052	0.215	0.202	0.085	0.046	0.0052	0.0049
LJ	0.002	0.0015	0.001	0.0004	0.0003	0.0001	0.0001	0.00006	0.0012	0.0009	0.002	0.0016	0.0001	0.00006	0.0063	0.0055
LK	0.005	0.0037	0.001	0.0005	0.0005	0.00022	0.0001	0.00005	0.0144	0.0102	0.064	0.047	0.0001	0.00005	0.0016	0.0013
LL	0.114	0.096	0.002	0.0009	0.0071	0.0028	0.0003	0.00012	0.111	0.092	0.195	0.177	0.021	0.016	0.0037	0.0032

^a^Non COVID-related lockdown time.

^b^COVID-related lockdown time.

**Figure 3:**
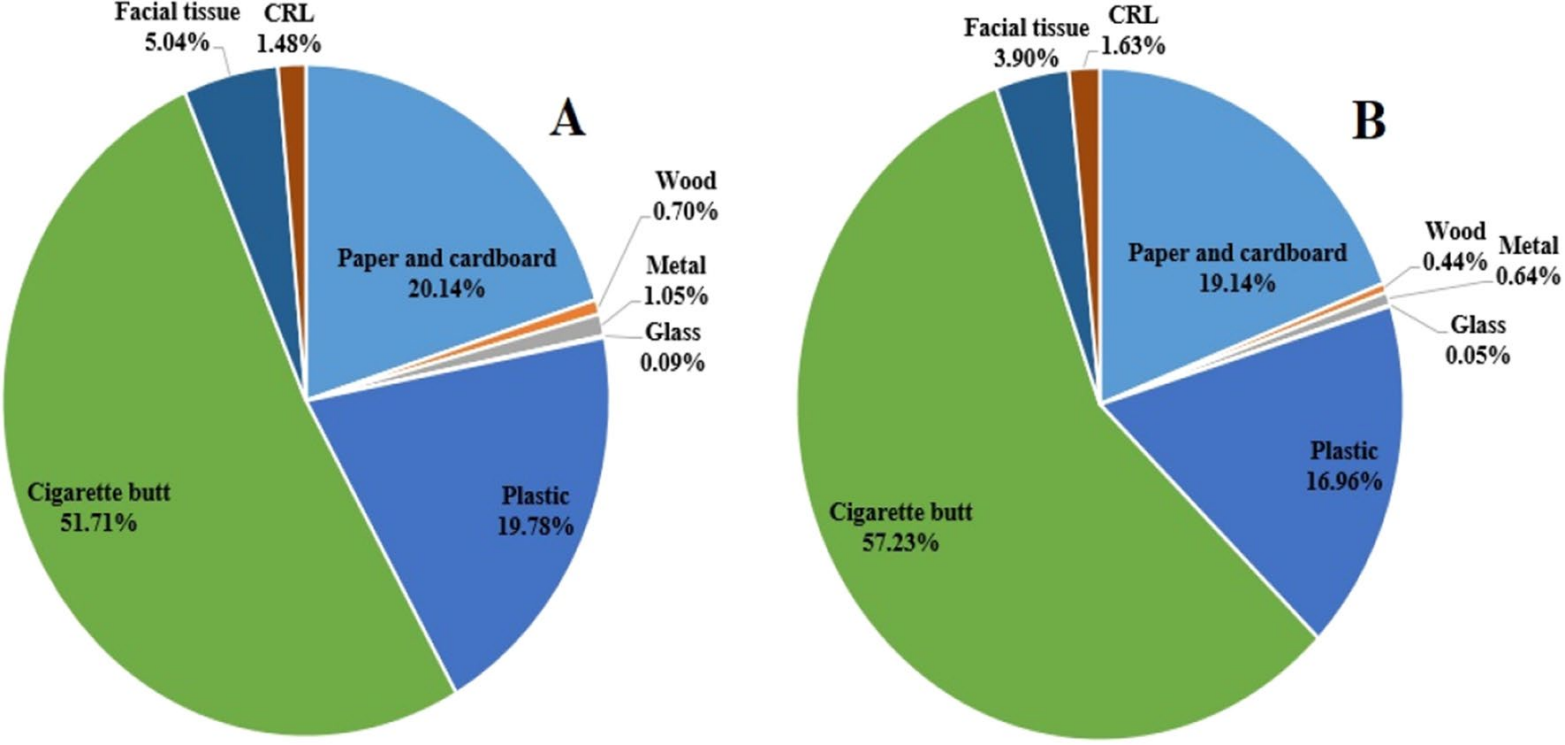
Composition of litter number in areas studied in non COVID-related lockdown time periods (**A**) and COVID-related lockdown time periods (**B**).

During this study, the two times COVID-related lockdown were implemented by the government, and the results of the litter assessment in the areas studied in these two periods showed that the litter density was reduced by an average of 19.86%. As shown in Fig. 3, the CRL density at the time of the lockdown was 0.001–0.0064 item/m^2^, while the density of CL was 0.0036–0.6060 item/m^2^. These conditions showed that during the COVID-related lockdown, the density of CL in the urban environment decreased by an average of 19.92%. The proportion of CRL in the total litter at this time was 1.63%, but under normal conditions was 1.48%. Therefore, spatial variation of litter density can be observed in the studied areas. Of course, during the pandemic, the trend of changes in the amount of litter is different from household waste. During the pandemic, due to the increase in online shopping, the amount of plastic in household waste increases^10^, but as shown in Fig. 3 owing to the decrease in the presence of people in public places and the outdoor environment, the amount of litter compounds decreases, but the proportion of plastic in them increases due to the increased use of face masks^29^.

Other studies have reported that litter is a major pollutant in many urban areas. In a city in Argentina, the density of litter was studied in four different areas, which showed that there was different litter densities in all areas and cigarette butt was the most CL^23^. In our study, the average density of cigarette butt was 0.153 number/m^2^, which was the highest CL. However, the composition of the litter in our study was different from other cities, so the highest numbers of litter were cigarette butts, facial tissue, paper, and plastic, respectively. One of the most important reasons for the difference in litter density in different urban areas can be the difference in land use^18^. In this study, the highest litter density was seen in commercial areas in LA, LD, and LG, while the recreational areas including LC, LH, and LJ had the lowest litter density. This difference was particularly noticeable in the case of CRL. As mentioned in Table 1, the highest CRL was observed in LC and LH, which were places of recreational areas, while the lowest density of CRL was observed in residential areas, including LB, LF, and LK. Differences in litter density in different land-uses mentioned in the results of previous studies^18,29,31^. One of the most important reasons for spatial variation is the impact of land use on population density^32^. In commercial areas, due to the higher people density, waste littering by citizens are more probably, and therefore the litter density in these areas will be higher than in other uses such as residential areas^23^. Moreover, some structural conditions in urban environments can cause more durability of the litter and thus increase the litter density in the environment. For example, the presence of low-access points such as tree pits, surface water collection canals, and bicycle stations reduces cleaning efficiency and increases litter density^31^. This situation was also observed in the case of CRL so that higher population density in commercial areas was one of the reasons for the higher density of CRL in these areas.

Littering potential of some types of municipal solid waste at specific points another reason for spatial variation is the litter density in the urban environment. For example, littered cigarette butts are more found around cigarette sales and consumption centers^20^ and more littered paper receipts are observed around banks and ATMs^18^. The difference in the number of these points in different parts of the city causes the variation of litter density. However, the results showed that the CRL density did not depend on this factor and no specific points were observed for higher density of this type of litter. The density of some types of litter such as cigarette butts around stalls and supermarkets and paper receipts around ATMs was higher than that in other areas. Also, the density of litter was not the same in the locations studied. Places, where people may stop for a while, have the potential for more litter density, the most important of which are intersections and urban transportation stations^18,25^. Differences in the quality of cleanup in different places can also be considered as a reason for spatial variation of litter density^32^. In this study, recreational areas had a different cleanup process from other places and therefore the litter density in them was different from other areas.

**Figure 4:**
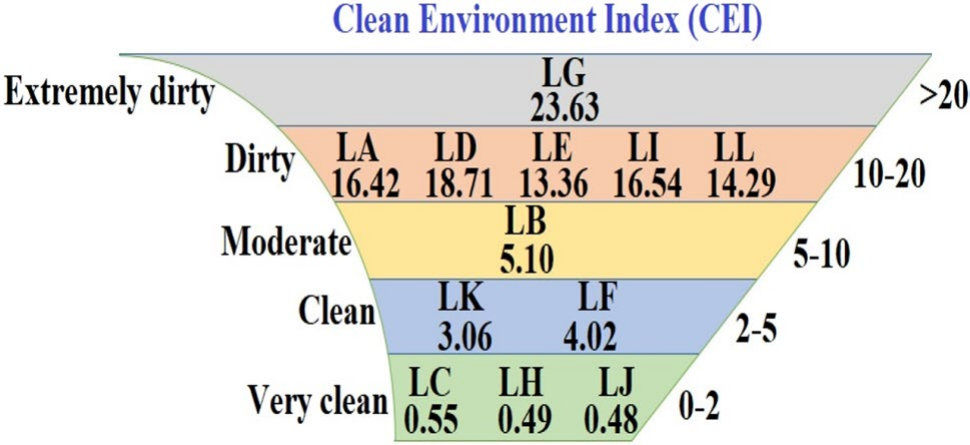
Calculated CEI for the locations studied.

The interpretation of urban environmental pollution status based on CEI is shown in Fig. 4. The results showed that 25% of the studied areas were in very clean status and 50% were in dirty and extremely dirty status. The average index for the studied areas was 9.72 and showed a moderate status. The use of CEI showed that this index shows the conditions in a more appropriate way compared to the litter density. Applying a coefficient for each litter and considering the importance of each litter in terms of pollution emission or environmental risk ranked the impact of each dump in the index. Among the litter, cigarette butt had the most important effect on CEI due to their large number and coefficient equal to 2. This index covers the effect of the type of litter, but it does not reflect the amount of pollutant leakage from different litter in different climatic conditions^30^. Due to the fact that cigarette butts contain various pollutants such as toxins^33^, metals^34^, and organic compounds^35^, a coefficient of 2 was applied for it. One of the important features of cigarette butts is owing to their rapid pollutant leakage into the environment, which makes this litter more important than other types. For example, it has been reported that nicotine leaks quickly from cigarette butts, and, in turn, the leaked nicotine can pollute one cubic meter of water^31^.

However, in this study, CRL was another important type of liter that had a significant impact on CEI. This type of litter is made of plastic-based materials and is known as a source for microplastic^36^ as well as the possibility of virus infection. Hence, CRL was applied in the index by a coefficient equal to 2.5, which was the highest coefficient among the types of litter. As Table 2 shows, the impact of each type of litter on CEI was different for the locations studied. This effect was dependent on the quantity of litter and the coefficient of each litter.

**Table 2:** Effect of litter types on calculated CEI for areas studied (%).

	Paper and cardboard	Wood	Metal	Glass	Plastic	Cigarette butt	Facial tissue	CRL
LA	14.45	0.33	0.86	0.042	18.51	60.16	4.28	1.33
LB	2.023	0.35	0.15	0.03	9.58	86.56	0.14	1.14
LC	3.50	3.31	0.32	0.65	4.07	14.12	0.68	73.32
LD	12.07	0.19	0.61	0.02	14.61	67.06	4.56	0.83
LE	14.38	0.41	0.61	0.04	25.32	52.80	4.90	1.50
LF	2.32	0.45	0.09	0.08	11.16	84.36	0.18	1.32
LG	10.77	0.38	0.29	0.03	16.35	68.54	2.27	1.33
LH	7.62	3.77	0.74	1.90	5.16	7.82	2.30	70.66
LI	12.56	0.44	0.72	0.02	14.26	51.44	18.97	1.55
LJ	7.86	3.69	1.09	0.38	7.07	15.86	0.76	63.25
LK	3.12	0.59	0.29	0.05	13.41	79.85	0.11	2.52
LL	15.53	0.25	0.89	0.03	22.63	53.73	5.64	1.26

**Figure 5:**
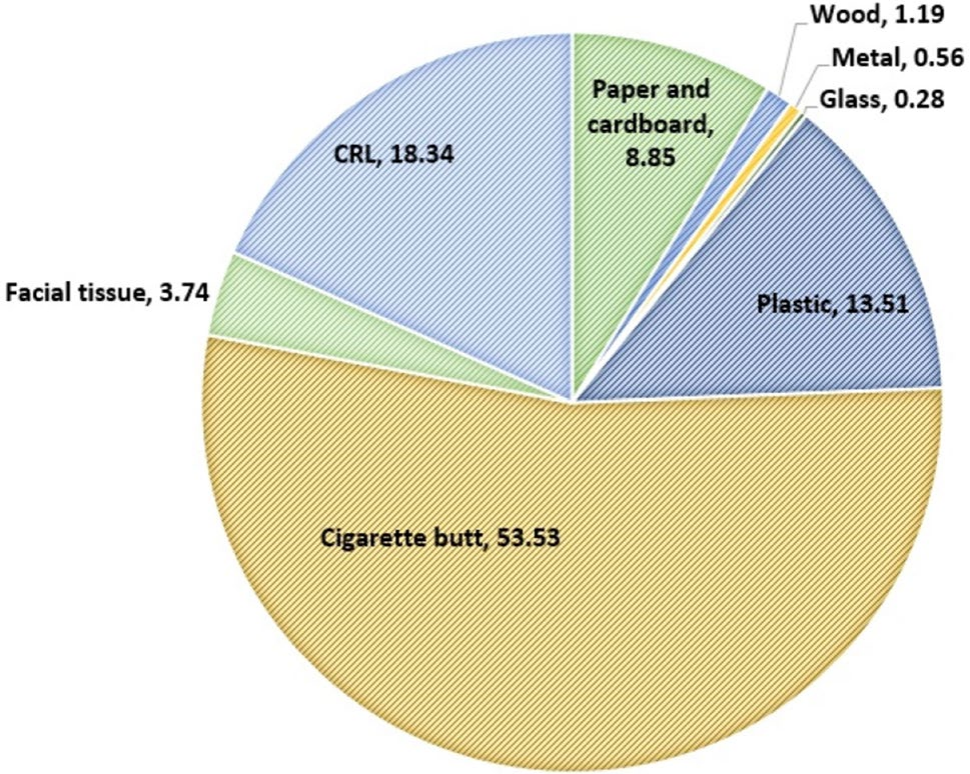
Average effect of litter types on calculated CEI.

CRL had an independent effect of 0.56–62% on the density of total litter in different locations in the urban environments, as well as the impact of these litter on the CEI of the areas studied was 1.2–73%, which averaged 18.34% (Fig. 5). A comparison of the periods with COVID-related lockdown with other days of the year showed that the pandemic led to change in litter composition in the studied locations on average (see Fig. 3). However, the significant impact of CRL on CEI showed that the pandemic due to the emergence of new types of litter in the urban environments resulted in an increase of 0.05–0.34 in the index score for the different locations studied. For this reason, the pandemic increased the pollution index score by an average of 0.21 point (the average ratio of CRL in the CEI were calculated for the studied locations as shown in Fig. 4).

Although the COVID-19 pandemic changed the density of litter in the urban environment and led to the emergence of new types of litter that can affect the landscape of the city, a more serious consequence of the pandemic to the urban environment is the possibility of damage due to degradation of CRL. Face masks and gloves were the two main CRLs which were observed in this study that constituted 1.5% of the total litter composition in the urban environments and were effective in the pollution index on average 18%. According to reports, in Iran, consumption of face masks and gloves during the COVID-19 pandemic was increased 55 times and 2.5 times, respectively^37^. The generation of waste from the consumption of this equipment, part of which is littered in cities, is a source of different kinds of microplastic in the environment that is a serious concern^38^. On the other hand, according to health protocols, the collection and disposal of these COVID-related wastes should be in separate bags^37^, but their littering in public places, as well as disposal with other municipal waste, cause the risk of infection transmission. This is especially the case for informal waste management staff in developing countries because most of these people do not use personal protective equipment during direct contact with municipal solid waste^39^.

In Iran, the management of medical wastes is the responsibility of its producer. For this reason, health centers and hospitals were equipped with disinfection devices such as autoclaves, which were able to manage well the COVID-related wastes during the pandemic^16^. However, in the case of CRL, a separate management system was not foreseen^37^ and the results of this study can be used in making decisions to improve the management of CRL. Given the impact of citizens' behavior on littering solid wastes in public places, efforts to improve citizens' behavior can be effective in reducing CRL^29^. This is especially important for face masks and gloves because there are special protocols for disposing of this waste during the pandemic^37^. One of the reasons for the presence of litter in public areas such as beaches can be the lack of trash^18^, however, in the urban areas studied in this study, the presence of many trash bins, a significant density of litter was seen. The situation in the areas studied showed that in the continuation of the COVID-19 pandemic and in probably similar situations in the future, the CRL should be better managed. In general, litter control in urban environments can be done in three phases: prevention, mitigation, and removal^21,40^. In the prevention steps by modifying the behavior of citizens through education and also applying anti-littering laws, litter can be reduced, including CRL. The most important action in the mitigation step is to reduce the density of litter, including CRL, the installation of trash bins in public places, as well as the installation of containers for CRL at specific intervals. Finally, the last step is to improve the cleanup efficiency of the urban environment, especially by identifying low access points in the removal step. Improving the management of litter can reduce the adverse consequences of landfilling, such as the risk of disease transmission^41,42^.

## Limitations and strengths of study

In this study, all land-uses were investigated and several locations from each land-use were studied. This was a field study and the data were obtained directly from the urban environment and represented the reality. A new index was used to interpret the data and the impact of the Covid-related litter was surveyed in this index. But this study had limitations such as the impossibility of considering all the streets of the city. Also, there was not enough time to study other cities and compare the results. Moreover, investigation the impact of litter on the pollution of water and soil resources and interpreting it by the new index was another drawback of the current research, which can be considered in future research.

## Conclusion

The effect of the COVID-19 pandemic on litter and urban pollution was investigated. The results showed that the COVID-related litter accounted for an average of 1.49% of the total solid wastes. Cigarette butt was the most common waste, accounting for 51.5% of the urban wastes. Also, 50% of the areas studied with a score of 10 and higher were in a dirty and worse status considering the CEI. Furthermore, 25% of the studied locations were in a very clean status considering the clean environment index. The COVID-related litter had a 1.2–73% effect on the pollution index, increasing the CEI by an average of 0.21 points. Although the COVID-19 pandemic reduced population density in public places and reduced the number of litter due to lockdown, it led to the emergence of new types of litter, resulting in an increase in the pollution index in the urban environments.

## Data Availability

All data generated or analyzed during this study are included in this published article.
